# Improving ESD Protection Robustness Using SiGe Source/Drain Regions in Tunnel FET

**DOI:** 10.3390/mi9120657

**Published:** 2018-12-12

**Authors:** Zhaonian Yang, Yuan Yang, Ningmei Yu, Juin J. Liou

**Affiliations:** Shaanxi Key Laboratory of Complex System Control and Intelligent Information Processing, Xi’an University of Technology, Xi’an 710048, China; yangyuan@xaut.edu.cn (Y.Y.); yunm@xaut.edu.cn (N.Y.); eenian2@126.com (J.J.L.)

**Keywords:** band-to-band tunneling (BTBT), electrostatic discharge (ESD), tunnel field-effect transistor (TFET), Silicon-Germanium source/drain (SiGe S/D), technology computer aided design (TCAD)

## Abstract

Currently, a tunnel field-effect transistor (TFET) is being considered as a suitable electrostatic discharge (ESD) protection device in advanced technology. In addition, silicon-germanium (SiGe) engineering is shown to improve the performance of TFET-based ESD protection devices. In this paper, a new TFET with SiGe source/drain (S/D) regions is proposed, and its ESD characteristics are evaluated using technology computer aided design (TCAD) simulations. Under a transmission line pulsing (TLP) stressing condition, the triggering voltage of the SiGe S/D TFET is reduced by 35% and the failure current is increased by 17% in comparison with the conventional Si S/D TFET. Physical insights relevant to the ESD enhancement of the SiGe S/D TFET are provided and discussed.

## 1. Introduction

A traditional metal-oxide-semiconductor field-effect transistor (MOSFET) has a 60 mV/dec subthreshold swing at room temperature, which limits the application of this device in ultra-low power integrated circuits (ICs) [1,2]. The tunnel field-effect transistor (TFET) is a promising candidate for replacing the conventional MOSFET in low power ICs [3,4,5]. The TFET employs a band-to-band tunneling (BTBT) mechanism and is able to theoretically achieve a subthreshold swing smaller than 60 mV/dec. However, the TFET has a very low driving current compared with the MOSFET, which means it is difficult to realize a high-speed circuit using pure TFETs. Recently, the mixed TFET–MOSFET circuit design methodology was reported, by skillfully designing the circuits such as static random access memory (SRAM), level shifter, and even electrostatic discharge (ESD) protection circuits with two kinds of devices, where both high performance and low standby current can be achieved [6,7,8,9]. ESD protection is a very challenging reliability issue of modern integrated circuits (ICs), especially in advanced nanoscale technologies [9,10,11,12,13,14]. As mentioned in reference [9], TFET can be used to replace the traditional diodes in an ESD protection network to enhance the ESD robustness in nanoscale technology ICs. The ESD behavior of the TFET has been studied using experiments and technology computer aided design (TCAD) simulations [15,16,17,18]. However, these results show that the ESD robustness of TFET under positive ESD stress is low.

It has been verified that using a silicon-germanium (SiGe) source in the TFET can increase the driving current compared with the silicon TFET [19,20,21,22]. This is because Ge has a narrower band-gap and lower carrier effective mass than Si, and these features increase the tunneling probability. The SiGe engineering has also been introduced in the ESD protection devices to enhance the ESD performance [12,13]. However, as for ESD protection applications, the physical processes mainly occur on the drain side of the TFET. As such, using a SiGe source does not benefit TFET’s ESD characteristics [16].

In this paper, we propose a new TFET with SiGe both in the source and drain (S/D) regions for ESD protection. The performance of the proposed device will be investigated using TCAD simulations. The simulation results will show that both the triggering voltage and the failure current of the SiGe S/D TFET are improved over those of the conventional Si TFET. The impact of various technology parameters on the ESD behavior of the SiGe S/D TFET will also be given.

## 2. Basic Concept of Electrostatic Discharge (ESD) Protection Tunnel Field-Effect Transistor (TFET) and the Protection Network

**Figure 1:**
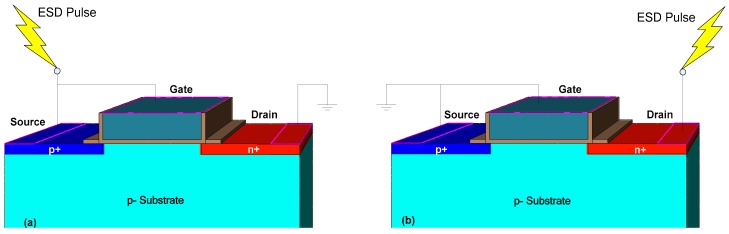
Schematics of tunnel field-effect transistor (TFET) under (**a**) negative and (**b**) positive electrostatic discharge (ESD) stresses.

TFET is essentially a reverse biased gated p-i-n diode. As for ESD protection, TFET, the gate terminal is connected to the source by default. Under the negative ESD stress, namely, ESD current is injected into the source terminal of TFET the with drain terminal grounded. TFET will operate in a positive diode conduction mode and has a high current discharge capability as illustrated in Figure 1a. Whereas under positive ESD stress, the ESD current is injected into the drain terminal with the source terminal grounded. TFET will operate in avalanche breakdown mode to discharge the ESD current as illustrated in Figure 1b. Since avalanche breakdown requires a relatively high electric field, the conduction voltage of TFET under positive ESD stress is high, making it unacceptable in advanced nanoscale technologies. Thus, the research on TFET under ESD stress mainly focuses on the positive discharge mode.

**Figure 2:**
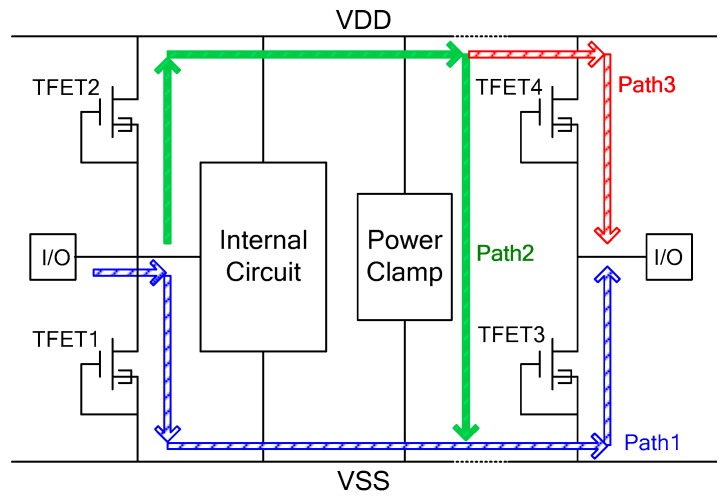
Schematic of the ESD protection network with TFETs.

It should be mentioned that, since TFET has a relatively low positive mode ESD robustness, it cannot be used as a single protection device in an IC, but can be used to implement a protection network as shown in Figure 2, in which TFET is used to replace the traditional diode to enhance the whole chip ESD robustness. As for the pin-to-pin ESD event, their discharge paths exist in the TFET based protection network as shown in Figure 2, whereas in the traditional diode-based protection network only Path2 exists. In Path1 and Path3, TFET1 and TFET4 operate in avalanche breakdown mode with low ESD robustness. Thus, it is necessary to improve the robustness of TFET under positive ESD stress.

## 3. Device Structure and Simulation Setup

**Figure 3:**
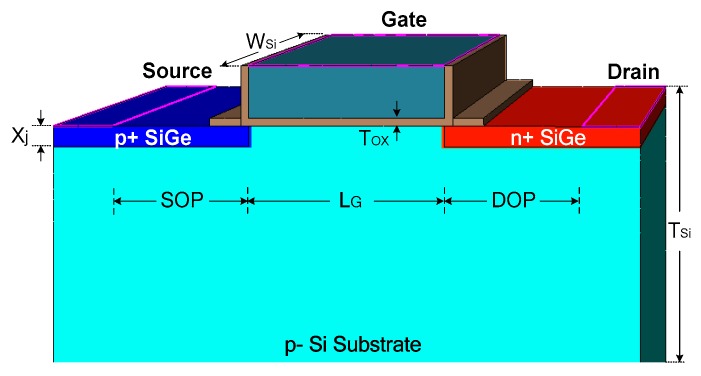
Schematic of the proposed SiGe source/drain (S/D) TFET.

As illustrated in Figure 3, the device structure proposed in this work is identical to the conventional silicon point-tunneling TFET except that the source and the drain regions are made of SiGe. The device size is not set to a very small value for better heat dissipation [11,23]. The default device parameters are: Thickness of the gate oxide (HfO_2_) T_ox_ = 4 nm, thickness of the silicon T_Si_ = 1 μm, width of the device W_Si_ = 1 μm, depth of the junction X_j_ = 10 nm, length of the gate L_G_ = 100 nm, and source and the drain side silicide blocking lengths SOP = DOP = 100 nm. Silicide blocking is used in ESD protection devices to reduce the current crowding effect [12,16]. The doping concentrations of the source, drain, and substrate are N_S_ = 1 × 10^20^ cm^−3^, N_D_ = 5 × 10^19^ cm^−3^, and N_Sub_= 1 × 10^16^ cm^−3^, respectively. Abrupt doping profile is used in the simulation. In order to avoid possible high defect density at the SiGe/Si interface, the default Ge mole fraction is set at 0.4 [22].

The SiGe S/D TFET can be fabricated using the following process flow. First, the source region is recessed into the p-Si substrate by an etching process. Then the p+ SiGe source region is grown by epitaxy. Similarly, the drain region is recessed into the p-Si substrate by the etching process and the n+ SiGe drain region is grown by epitaxy. Afterward, the gate dielectric and the gate stack are deposited and patterned. Finally, the spacers are formed.

Simulations are carried out in the Sentaurus simulator. The dynamic nonlocal BTBT model is used instead of the local BTBT model. This is because the dynamic model takes into account the spatial variation of the energy bands and therefore can model the BTBT probability more accurately. The fitted coefficients of the SiGe BTBT probability are calculated by linear interpolation between the parameters of pure Si and pure Ge [23]. The lattice temperature is calculated using the thermodynamic model. Van Overstraeten-de Man avalanche generation model, high field saturation, and Philips unified mobility models, band-gap narrowing model and doping dependent Shockley-Read-Hall recombination model are also used.

Transmission line pulsing (TLP) pulses, which mimic the stressing of the human body model (HBM), are used to simulate the quasi-static current-voltage (*I-V*) behavior of the devices during the ESD conditions. The drain terminal of the TFET was stressed with TLP pulses while keeping the gate and the source terminals grounded. The rise time and the pulsewidth are set at 10 ns and 100 ns, respectively. The voltage samples are obtained by averaging the transient data in the range of 60 ns to 90 ns [16].

## 4. Simulation Results and Discussion

**Figure 4:**
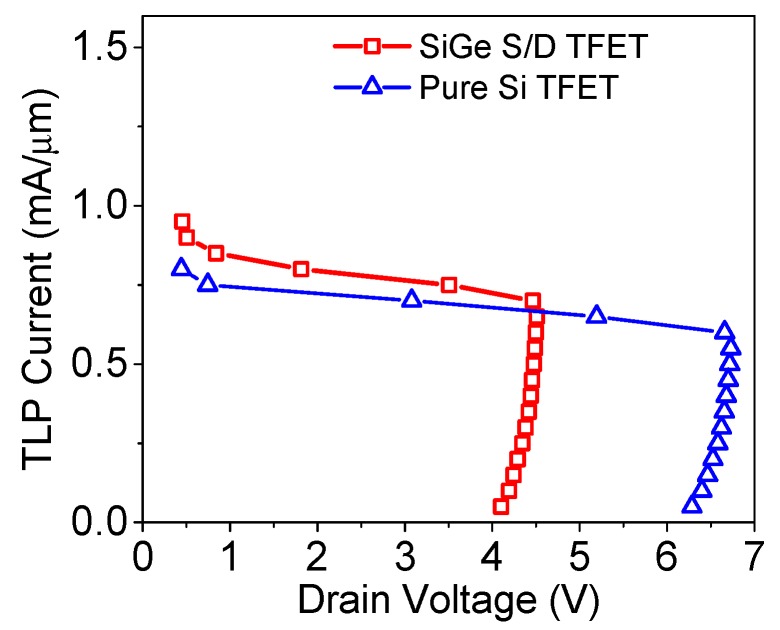
Transmission line pulsing (TLP) *I-V* curves of the SiGe S/D TFET and Si TFET.

The TLP *I-V* curves of both the SiGe S/D and Si TFETs are shown in Figure 4. The triggering voltage and failure current of the SiGe S/D TFET are 4.1 V and 0.7 mA/μm, respectively, which are 35% lower and 17% higher than those of the Si counterpart. These improved key parameters will make the new TFET easier to fit into the modern ESD design window and offer higher ESD protection capability. It should be noted that the SiGe source has nearly no influence on the ESD characteristics [15], and the improvement is achieved by introducing the SiGe drain in the TFET.

**Figure 5:**
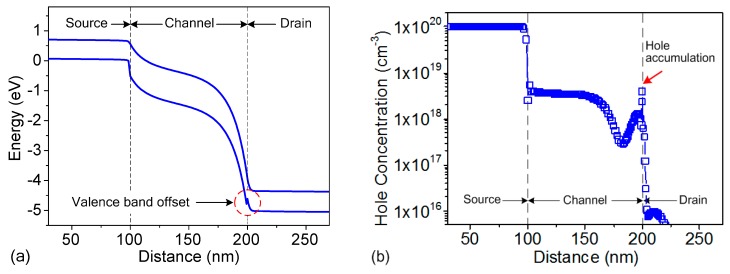
(**a**) Energy bands and (**b**) hole concentration simulated at 90 ns under a TLP current density of 0.5 mA/μm, at a distance of 5 nm below the Si/SiO_2_ interface.

The reduction of the triggering voltage of the TFET is achieved by introducing the SiGe material in the drain region. The Ge material has the following three advantages in triggering the TFET at a lower voltage. First, Ge has a higher BTBT probability than Si due to its narrower bandgap and lower carrier effective mass. The TFET has a BTBT-assisted avalanche generation mechanism, hence a higher BTBT probability gives rise to a more significant avalanche breakdown [16,17]. Second, Ge has a higher impact ionization coefficient than Si under the same electric field [24]. This means that the critical electric field required for avalanche breakdown in the SiGe S/DTFET is lower than that in the Si TFET. Third, the drain/substrate heterojunction offers an enhanced electric field, which helps to reduce the triggering voltage [25]. SiGe and Si have similar electron affinities, thus the bandgap difference approximately equals the valence band offset. Figure 5a shows the energy bands of the SiGe S/D TFET stressed under a TLP current density of 0.5 mA/μm. It can be seen that there is a valence band offset at the drain/substrate interface. This obstructs the holes from moving to the source, causing some holes to accumulate on the drain side, as evidenced by the hole concentration plot shown in Figure 5b, with a significant hole density peak at a distance of 5 nm below the Si/SiO2 interface on the drain side. This leads to an enhancement in the electric field at the drain/substrate interface and consequently a reduction in the trigger voltage.

**Figure 6:**
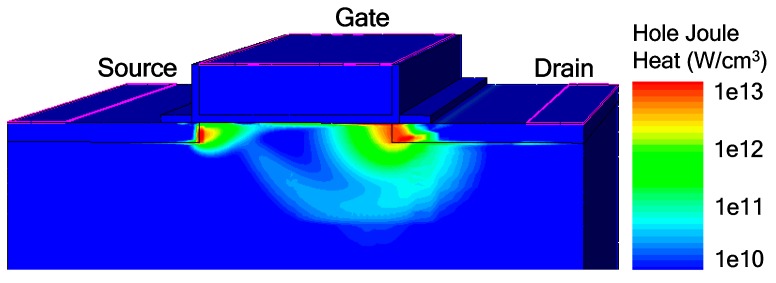
Contour plot of hole Joule heat simulated at 90 ns under a TLP current density of 0.5 mA/μm.

**Figure 7:**
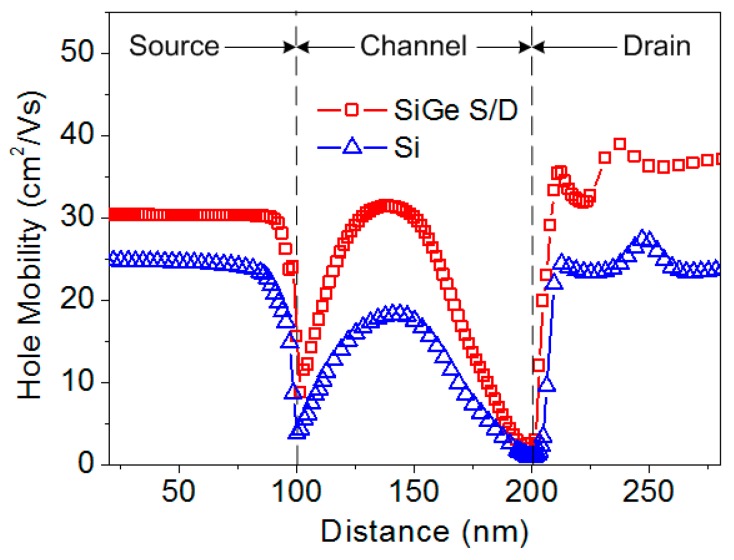
Hole mobilities at a distance of 5 nm below the Si/SiO_2_ interface.

As shown in Figure 2, the failure current of the new TFET is also improved. Under an ESD event, the Joule heat is the main heat component in the device, and it can be expressed as in reference [26],

(1) $$H_{\mathrm{Joule}}=H_{p}+H_{n}=\frac{|J_{p}{|}^{2}}{pq\mu_{p}}+\frac{|J_{n}{|}^{2}}{nq\mu_{n}}$$

where *H* is the heat, *J* is the current density, *μ* is the mobility, and subscripts *n* and *p* denote electrons and holes, respectively. The hole Joule heat is higher than the electron Joule heat because the impact generated holes move from the drain interface to the source through the channel region, whereas the electrons are collected by the drain terminal without traveling. Furthermore, the high electric field and carrier scattering significantly degrade the mobility, especially near the drain and the source interfaces. These, in turn, cause a large amount of hole Joule heat generated at the interface regions as shown in Figure 6. The hole mobility in the SiGe S/D TFET is higher than that in the conventional Si TFET as shown in Figure 7. Thus, the SiGe S/D TFET has an elevated robustness due to the fact that the hole Joule heat is the dominate heat source and hole mobility in SiGe is higher than that in Si.

The thermal conductivity is another important factor influencing the ESD thermal breakdown. SiGe has a lower thermal conductivity compared with Si, which hinders the heat dissipation [27,28]. However, the volume of the SiGe regions are relatively small and the reduction in the triggering voltage implies that less Joule heat is generated.

**Figure 8:**
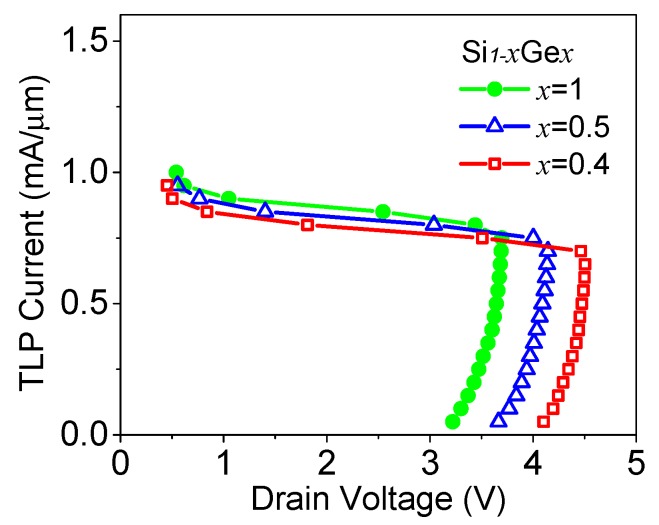
TLP *I-V* curves of SiGe S/D TFET with different Ge mole fractions.

In the SiGe S/D TFET, the increase in the Ge mole fraction (*x*) can cause a reduction in the triggering voltage, and a slight increase in the failure current as shown in Figure 8. This trend can be easily understood from the preceding discussions. However, when the Ge mole fraction is higher than 0.4, the defect density at the SiGe/Si interface may degrade the device performance.

**Figure 9:**
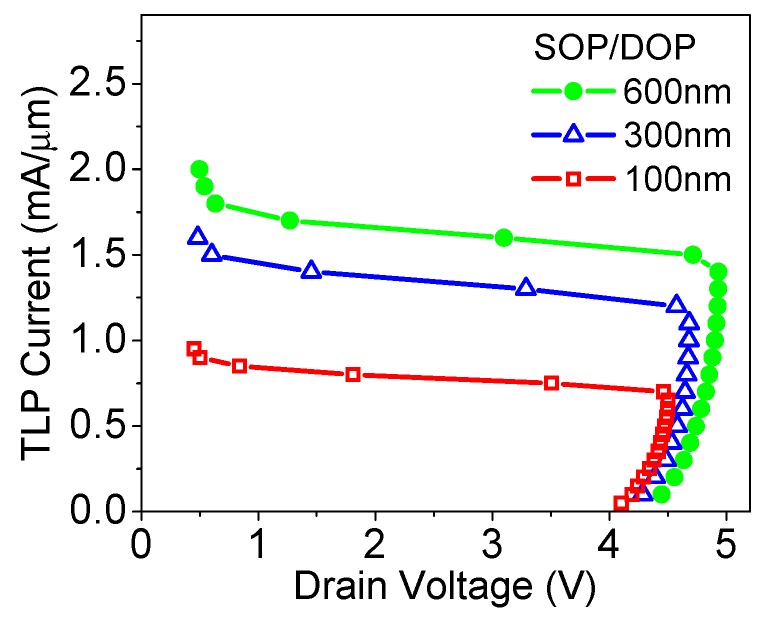
TLP *I-V* curves of SiGe S/D TFET with different SOP and DOP values.

**Figure 10:**
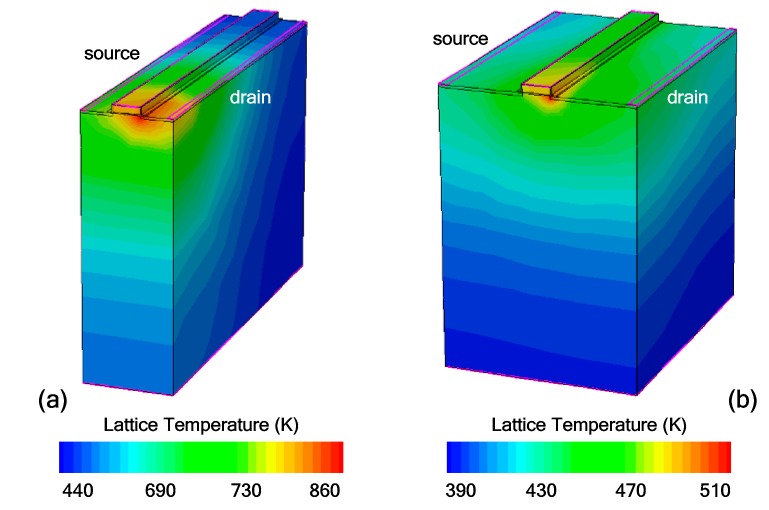
Contour plots of lattice temperatures in TFETs under TLP current density of 0.5 mA/μm with different DOP/SOP values: (**a**) DOP = SOP = 100 nm and (**b**) DOP = SOP = 300 nm.

Dimensions have significant influences on the characteristics of ESD protection devices. From Figure 9, it can be seen that with a large DOP and SOP value (see Figure 1), although the triggering voltage is slightly increased, the failure current is significantly increased. This can be attributed to two reasons. The increase in device volume offers a better heat dissipation and thus a reduced temperature in the device. In addition, when DOP and SOP are increased, the series resistance in the discharge path is increased, hence the ballasting effect suppresses the current crowding along the lateral direction [16]. The contour plots of lattice temperature with two DOP and SOP values are shown in Figure 10. 

The gate length can also affect the ESD performance. As listed in Table 1, the scaling in the gate length reduces the triggering voltage and the failure current of the TFET. The former can be attributed to the increase in the lateral electric field, which enhances the reverse biased p-n junction tunneling and impact ionization [29]. In addition, the increase in spreading resistance may also play a role [30,31]. However, since the gate is grounded, the electric field near the drain/substrate junction is strongly affected by the gate, and the impact of gate length on the triggering voltage is not very significant [18]. The failure current increases with increasing gate length owning to the larger size and improved conduction uniformity.

**Figure 11:**
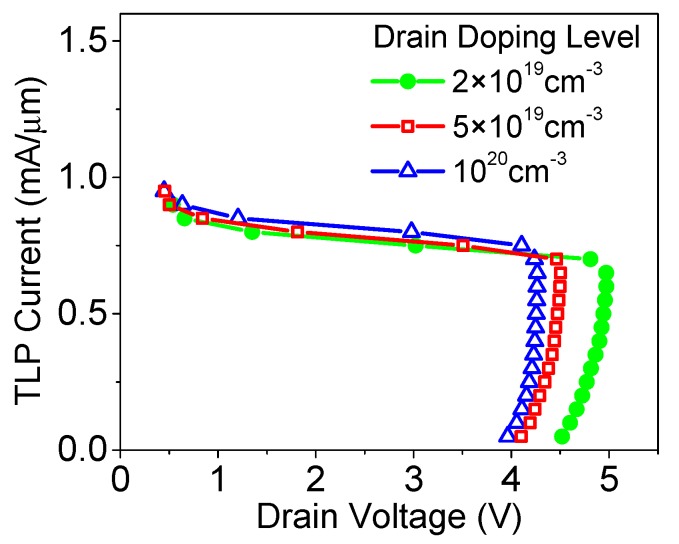
TLP *I-V* curves of SiGe S/D TFET with different Ge mole fractions.

The impact of drain doping level on SiGe S/D TFET’s ESD *I-V* characteristic is shown in Figure 11. It can be observed that with the increase in drain doping level, the triggering voltage is reduced. This can be attributed to the enhanced BTBT, and reduction in the critical electric field required for avalanche breakdown. The failure current is slightly increased with the increase in drain doping level, and this is because the reduction in drain voltage results in less Joule heat. It should be mentioned that, since the BTBT and avalanche generations mainly occur on the drain side, the source doping level nearly does not influence the TFET’s ESD characteristics [16].

## 5. Conclusions

In this paper, a new grounded-gate TFET with SiGe source and drain regions was proposed and its ESD characteristics were investigated using TCAD simulations. Compared to the conventional Si TFET, the triggering voltage of the SiGe S/D TFET is reduced because the SiGe regions offer a high BTBT probability, a higher impact ionization coefficient, and a higher electric field due to the SiGe/Si heterostructure. The failure current of the SiGe S/D TFET is also increased due to the combination of a lower triggering voltage and a smaller Joule heat resulting from a higher hole mobility in SiGe. This enhanced ESD performance will be beneficial for constructing robust TFET-based ESD protection networks in the future.

## Figures and Tables

**Table 1:** Triggering voltages and failure currents with different gate lengths.

Gate Length	50 nm	100 nm	150 nm	200 nm
Triggering Voltage	4.06 V	4.1 V	4.18 V	4.28 V
Failure Current	0.65 mA/μm	0.7 mA/μm	0.725 mA/μm	0.75 mA/μm
